# UNDERSTANDING THE CURRENT LOCATION DISPARITIES IN ADRD AND ITS DETERMINANTS IN NIGERIA

**DOI:** 10.1093/geroni/igad104.0519

**Published:** 2023-12-21

**Authors:** Oloaigbe (Joyce) Ogidigo, Okiemute Okpalefe, Kingsley Omeje, Chioma Anosike

**Affiliations:** University of Miami, Miami, Florida, United States; National Biotechnology Development Agency, Abuja, Federal Capital Territory, Nigeria; University of Nigeria, Nsukka, Enugu, Nigeria; University of Nigeria, Enugu, Enugu, Nigeria

## Abstract

Alzheimer’s disease and related dementias (ADRD) is a growing health challenge in low- and middle-income countries, particularly in sub-Saharan Africa. Evidence of geographical variation is associated with increased ADRD incidence, and current research suggests that early-life rural living further increases ADRD risk. However, the scarcity of studies conducted in resource-poor countries limits conclusions. Consequently, the awareness and response to ADRD have been limited. In Nigeria, several rural communities still associate dementia with the normal process of aging, with many patients stigmatized and abandoned in the belief that their condition is beyond any medical intervention. Thus, many of those affected delay seeking medical care and endure poor outcomes. This is exacerbated by the lack of mental health service access in rural areas. This geographic disparity and deeply rooted structural inequalities have posed additional challenges to adequately diagnose and provide care for individuals across the lifespan living in these settings. Hence, population-based data on ADRD incidence in Nigeria is required to estimate its true burden and inform the design of ADRD prevention and management strategies. We report our analysis of the current situation of ADRD in rural Nigeria while exploring future perspectives and directions. For example, heath policies to increase the ratio of mental health professionals per number of patients, mostly in rural areas, foment proactive primary care centers. Ultimately, understanding rural-urban variation in ADRD diagnostic incidence and prevalence will inform needed policies to improve timely management and access to supportive services for older adults in Nigerian and other under-resourced regions.

